# Body weight gain rather than body weight variability is associated with increased risk of nonalcoholic fatty liver disease

**DOI:** 10.1038/s41598-021-93883-5

**Published:** 2021-07-13

**Authors:** Eun Ju Cho, Su Jong Yu, Gu Cheol Jung, Min-Sun Kwak, Jong In Yang, Jeong Yoon Yim, Goh Eun Chung

**Affiliations:** 1 Department of Internal Medicine, Liver Research Institute, Seoul National University College of Medicine, Seoul, Republic of Korea; 2 Department of Internal Medicine, Healthcare Research Institute, Seoul National University Hospital Healthcare System Gangnam Center, 39FL., Gangnam Finance Center 737, Yeoksam-Dong, Gangnam-Gu, Seoul, 135-984 South Korea; 3 Healthcare Research Institute, Seoul National University Hospital Healthcare System Gangnam Center, Seoul, Republic of Korea

**Keywords:** Hepatology, Obesity

## Abstract

Weight loss, the most established therapy for nonalcoholic fatty liver disease (NAFLD), is frequently followed by weight regain and fluctuation. The aim of this study was to investigate whether body weight change and variability were independent risk factors for incident NAFLD. We conducted a longitudinal cohort study. Among the 1907 participants, incident NAFLD occurred in 420 (22.0%) cases during median follow-up of 5.6 years. In the multivariate analysis, there was no significant association between weight variability and the risk of incident NAFLD. The risk of incident NAFLD was significantly higher in subjects with weight gain ≥ 10% and 7% < gain ≤ 10% [hazard ratios (HR), 2.43; 95% confidence intervals (CI), 1.65–3.58 and HR, 1.73; 95% CI, 1.26–2.39, respectively], while the risk of incident NAFLD was significantly lower in those with −7% < weight loss ≤ -−3% (HR, 0.33; 95% CI, 0.22–0.51). Overall body weight gain rather than bodyweight variability was independently associated with the risk of incident NAFLD. Understanding the association between body weight variability and incident NAFLD may have future clinical implications for the quantification of weight loss as a treatment for patients with NAFLD.

## Introduction

Non‐alcoholic fatty liver disease (NAFLD) is the most common cause of chronic liver disease worldwide^1,2^. It encompasses a spectrum of progressive liver disease from simple steatosis to steatohepatitis, hepatic fibrosis, and cirrhosis^3^. Obesity, insulin resistance, type 2 diabetes, and dyslipidemia are the key risk factors for the development of NAFLD. Although there is no currently available treatment for NAFLD, there have been several studies showing that NAFLD is somewhat reversible. Changes in lifestyle habits such as weight reduction, and regular exercise were associated with remission of NAFLD^4^.

Body weight loss is the most established therapy for obesity-associated metabolic risk factors and NAFLD, with an evident dose-dependent association^5^. Accordingly, several guidelines recommend a 7–10% weight loss as the target of lifestyle modifications in overweight or obese NAFLD patients^6–8^. However, weight loss is frequently followed by weight regain or variability, with 80% of subjects who lose > 10% weight regaining it within one year^9^. Recent studies have reported that weight variability is associated with the increased risk of cardiovascular events and mortality in patients with established coronary artery disease^10^ or diabetes mellitus^11^. Furthermore, weight variability has been found to be associated with higher risk of incident type 2 diabetes in the general population^12,13^. However, few studies have investigated the association between weight variability and NAFLD. In addition, differences in its effects regarding baseline weight or change in weight have yet to be evaluated.

Therefore, the aim of the present study is to investigate whether body weight variability and weight change are independent risk factors for incident NAFLD in a health check-up population.

## Methods

### Study population

The study participants were part of a previous cohort study^14^. Briefly, the initial cohort for this study consisted of 34,080 subjects who had completed a comprehensive health check-up including abdominal ultrasonography and laboratory exams from January 2007 to February 2015 at Seoul National University Hospital Healthcare System Gangnam Center. Baseline and follow-up examinations were conducted annually or biennially between March 2008 and December 2017 at the same institute. Individuals who have at least one cause of chronic liver disease were excluded; positive for serum hepatitis B surface antigen, positive for antibody against hepatitis C virus, and/or a history of chronic liver disease as identified by a detailed questionnaire. Among them, we enrolled subjects who had undergone more than three exams with an interval of one year or more between each test.

The total number of eligible subjects for the study was 5636 at baseline. We excluded 2258 participants with NAFLD at the time of baseline and 1382 subjects with significant alcohol consumption (> 30 g/day for men and > 20 g/day for women)^7^. We further excluded 89 subjects with missing baseline information. As a result, a total of 1907 participants were included in the analysis. The study protocol was approved by the Institutional Review Board of Seoul National University Hospital (2003-122-1110) and confirmed to the ethical guidelines of the World Medical Association Declaration of Helsinki. As the study used de-identified secondary data, the requirement for informed consent from individuals was waived by the Institutional Review Board of Seoul National University Hospital.

### Clinical parameters and biochemical analysis

As previously described^15^, standardized self-reported questionnaires were used to collect data at the time of enrollment. Height and weight were measured using a digital scale. Body mass index (BMI) was calculated as weight (kg) divided by the square of the person’s height (m). Well-trained personnel measured the waist circumference (WC) at the midpoint between the lower costal margin and the iliac crest. Systolic and diastolic blood pressures were measured twice on the same day. Systolic/diastolic blood pressure ≥ 140/90 mm Hg and/or previous use of antihypertensive medication were used to define hypertension. Fasting glucose levels ≥ 126 mg/dL and/or an oral hypoglycemic agent or insulin treatment were defined as clinical presentations of diabetes mellitus.

After an overnight fast of ≥ 8 h, blood specimens were obtained from each participant. Laboratory tests included serum levels of total cholesterol, high-density lipoprotein (HDL) cholesterol, triglycerides, fasting glucose and alanine transaminase (ALT).

### Definition of body weight variability and change

Intra-individual body weight variability can be measured by various methods. Widely used indices in previous studies include standard deviation (SD), coefficient of variation (CV), average real variability (ARV), and variability independent of the mean (VIM)^16,17^. CV is calculated as the ratio of SD to the mean. ARV is defined as the average absolute differences between successive body weight measurements and reflects the order of measurements. VIM is a measure of variability that has no correlation with mean levels over visits. VIM is calculated as the SD divided by the mean to the power *x* and multiplied by the population mean to the power *x,* that is the regression coefficient based on the natural logarithm of the SD over the natural logarithm of the mean^17^. We divided the body weight variability indices into quartiles for analysis (Q1 = lowest quartile; Q4 = highest quartile). In addition, we categorized the overall body weight change (OBC) into three or seven groups to evaluate the direction of body weight variability; [loss ≥—5%, < -5% to < 5%, and gain ≥ 5%] and [loss ≥—10%, -10% < loss ≤ -7%, -7% < loss ≤ -3%, < -3% to < 3%, 3% < gain ≤ 7%, 7% < gain ≤ 10%, and gain ≥ 10%]^16^.

### Definitions of NAFLD

NAFLD was defined by the evidence of hepatic steatosis based on the characteristic ultrasonographic features without excessive alcohol consumption or concomitant liver disease^6,7^. Hepatic ultrasonography (Acuson Sequoia 512; Siemens, Mountain View, CA) was performed to diagnose fatty liver by experienced radiologists who were blinded to the clinical characteristics of the subjects. Fatty liver was diagnosed based on characteristic ultrasonographic features consistent with a ‘‘bright liver,’’ evident contrast between hepatic and renal parenchyma, vessel blurring, focal sparing, and luminal narrowing of the hepatic veins^18^.

### Statistical analyses

Data are presented as mean ± standard deviation for normally distributed, continuous variables and as proportions for categorical variables, unless otherwise indicated. Log transformations were performed for non-normally distributed variables. The comparison of baseline characteristics was conducted using independent t-tests and analysis of variance for continuous variables and the chi-square test for categorical variables. Among variables with a *P* value < 0.05 in univariate analyses, those with clinical importance were subjected to multivariate analyses. Cox-proportional hazard regression was performed to estimate the risk of incident NAFLD. To control for confounding, we adjusted the model for baseline body weight, age, sex, hypertension, diabetes mellitus, waist circumference, BMI, triglyceride, HDL-cholesterol, ALT, and number of measurements. Statistical analyses were performed using SAS version 9.4 (SAS Institute, Cary, NC, USA) and R version 4.0.4 (R Project for Statistical Computing, Vienna, Austria, http://www.Rproject.org). A two-sided P value < 0.05 was considered statistically significant.

## Results

### Baseline characteristics of the study population

The mean age of the study population was 50.8 years, and 47.6% was male. Among the total 1,907 subjects, incident NAFLD occurred in 420 (22.0%) cases during the median follow-up period of 5.6 years (interquartile range, 4.6–6.3). The mean number of weight measurements was 6.7 ± 3.1 and the body weight of each subject was measured 3 times (15.8%), 4 times (15.2%), or more than 5 times (69.0%).

The baseline characteristics of the subjects according to quartiles of weight variability are shown in Table 1. Higher weight variability was more frequently discovered in subjects who were younger and in males with higher waist circumference and BMI. Total cholesterol and HDL-cholesterol levels decreased with higher weight variability, whereas triglyceride showed an increasing trend with higher weight variability. Fasting glucose and ALT levels were not significantly different between the groups. Prevalence of diabetes and hypertension were also similar across the groups.

**Table 1:** Baseline characteristics of the study population according to quartiles of weight variability.

	Q1 (lowest)	Q2	Q3	Q4 (highest)	*P*
	N = 477	N = 480	N = 477	N = 473	
Age (years)	51.3 ± 8.9	51.3 ± 8.0	51.1 ± 8.6	49.3 ± 9.4	< 0.001
Male (number, percent)	206 (43.2%)	226 (47.1%)	217 (45.5%)	258 (54.5%)	0.003
Waist circumference (cm)	79.8 ± 7.2	81.6 ± 6.7	81.5 ± 7.2	82.8 ± 7.0	< 0.001
Body mass index (kg/m^2^)	21.8 ± 2.4	22.3 ± 2.3	22.3 ± 2.4	22.9 ± 2.5	< 0.001
**Weight change**					< 0.001
< -5%	0 (0.0%)	7 (1.5%)	34 (7.1%)	119 (25.2%)	
−5 to 5%	477 (100.0%)	451 (94.0%)	335 (70.2%)	154 (32.6%)	
≥ 5%	0 (0.0%)	22 (4.6%)	108 (22.6%)	200 (42.3%)	
**Comorbidity**					
Diabetes	21 (4.4%)	16 (3.3%)	15 (3.1%)	24 (5.1%)	0.380
Hypertension	44 (9.2%)	48 (10.0%)	53 (11.1%)	60 (12.7%)	0.340
**Laboratory findings**					
Fasting glucose (mg/dL)	92.1 ± 14.5	91.5 ± 13.2	91.2 ± 11.9	92.5 ± 16.1	0.820
Total cholesterol (mg/dL)	196.5 ± 33.3	191.6 ± 32.7	193.2 ± 32.1	189.2 ± 31.5	0.003
HDL cholesterol (mg/dL)	58.3 ± 13.4	56.1 ± 12.8	56.9 ± 12.2	54.7 ± 12.4	< 0.001
Triglyceride (mg/dL)	81.2 ± 43.9	85.0 ± 40.4	85.0 ± 43.7	90.6 ± 48.2	0.002
ALT (IU/L)	20.2 ± 10.3	21.0 ± 13.7	20.8 ± 12.0	21.3 ± 12.0	0.230
**Number of weight measurements**					0.004
3 (number, percent)	99 (20.8%)	71 (14.8%)	61 (12.8%)	71 (15.0%)	
4 (number, percent)	94 (19.7%)	56 (11.7%)	62 (13.0%)	77 (16.3%)	
5 (number, percent)	52 (10.9%)	50 (10.4%)	59 (12.4%)	55 (11.6%)	
6 (number, percent)	61 (12.8%)	68 (14.2%)	64 (13.4%)	53 (11.2%)	
More than 7 (number, percent)	172 (36.1%)	235 (49.0%)	231 (48.4%)	217 (45.9%)	

Data are presented as mean ± standard deviation for continuous variables and n (%) for categorical variables.

*Q* quartile, *HDL* high density lipoprotein, *ALT* alanine transaminase.

### Body weight variability and incident NAFLD

First, we evaluated the association of weight variability and incident NAFLD (Table 2). In the univariate analysis, most body weight variability indices including SD, CV and VIM were significantly associated with lower risk of incident NAFLD (p < 0.05). However, no significant association between weight variability and the risk of incident NAFLD after adjusting for age, sex, hypertension, diabetes, waist circumference, BMI, total cholesterol, triglyceride, HDL-cholesterol levels, ALT, and number of measurements. Similar results were observed when subjects were divided according to the direction of weight change metrics (Supplementary Table 1).

**Table 2:** The risk of incident NAFLD regarding level of body weight variability.

Body weight variability	No. of patients	No. of events	Univariate analysis			Multivariate analysis		
			Hazard ratio (95% CI)	P-value	Overall *P*-value	Hazard ratio (95% CI)	P-value	Overall *P*-value
**SD**								
Q1	477	81	1 (ref.)			1 (ref.)		
Q2	480	100	0.71 (0.53, 0.95)	0.02	0.03	0.75 (0.55, 1.01)	0.06	0.23
Q3	477	105	0.75 (0.56, 1.00)	0.05		0.77 (0.57, 1.04)	0.09	
Q4	473	134	0.94 (0.72, 1.25)	0.69		0.85 (0.64, 1.13)	0.27	
**CV**								
Q1	483	98	1 (ref.)			1 (ref.)		
Q2	481	103	0.71 (0.54, 0.94)	0.02	0.01	0.82 (0.62, 1.08)	0.16	0.31
Q3	469	103	0.64 (0.49, 0.85)	0.002		0.78 (0.59, 1.04)	0.09	
Q4	474	116	0.7 (0.54, 0.92)	0.01		0.8 (0.60, 1.06)	0.12	
**ARV**								
Q1	464	88	1 (ref.)			1 (ref.)		
Q2	470	97	0.87 (0.65, 1.16)	0.34	0.08	1.01 (0.75, 1.36)	0.94	0.90
Q3	492	120	1.03 (0.78, 1.35)	0.85		1.03 (0.78, 1.36)	0.85	
Q4	481	115	1.24 (0.94, 1.63)	0.13		0.93 (0.70, 1.24)	0.63	
**VIM**								
Q1	484	95	1 (ref.)			1 (ref.)		
Q2	478	101	0.7 (0.53, 0.93)	0.01	0.02	0.81 (0.60, 1.07)	0.14	0.39
Q3	470	104	0.66 (0.50, 0.88)	0.004		0.81 (0.61, 1.08)	0.16	
Q4	475	120	0.75 (0.57, 0.98)	0.04		0.82 (0.62, 1.08)	0.15	

Adjusted for age, sex, hypertension, diabetes, waist circumference, body mass index, triglyceride, high density cholesterol, total cholesterol, alanine transaminase, and number of measurements.

*NAFLD* nonalcoholic fatty liver disease, *CI* confidence interval, *SD* standard deviation, *CV* coefficient of variation, *ARV* average real variability, *VIM* variability independent of mean, *Q* quartile.

### Incident NAFLD risk according to overall weight change

**Figure 1:**
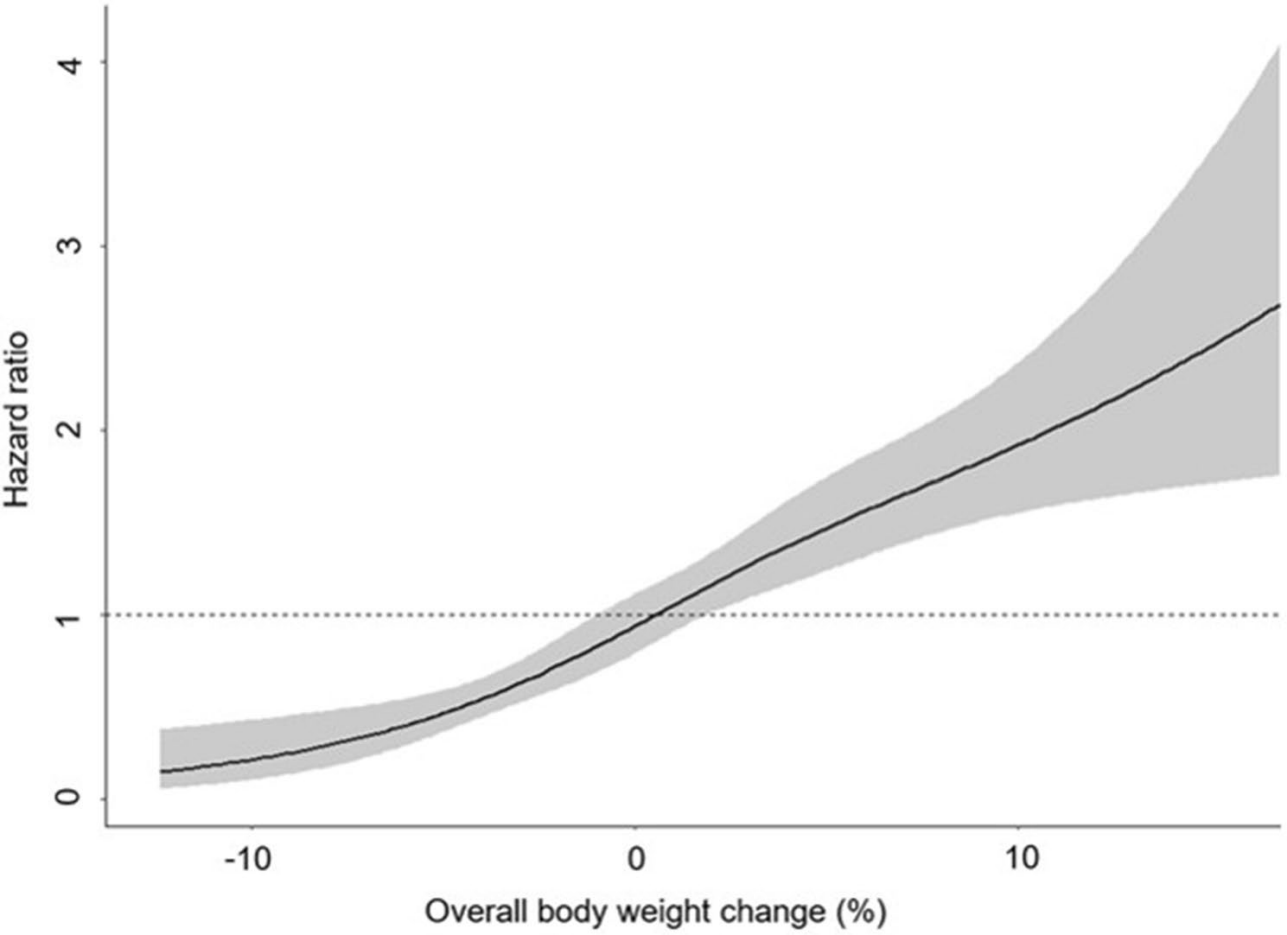
The hazard ratio plot showing the correlation between overall body weight change and risk of incident NAFLD. The models were fitted with restricted cubic splines with 4 knots placed at the 5th, 35th, 65th, and 95th percentiles of overall body weight change (model selection and knot placement via Bayesian information criterion) and the curves were adjusted for variables in a multivariate model including age, sex, hypertension, diabetes, waist circumference, body mass index, ALT, triglyceride, high density cholesterol and total cholesterol. NAFLD, nonalcoholic fatty liver disease; ALT, alanine transaminase.

Next, we evaluated the effect of OBC on the development of NAFLD. OBC per 10% increase was associated with the risk of NAFLD development (adjusted HR 2.28, 95% CI, 1.90–2.74). When the participants were categorized into three groups according to OBC, the risk of incident NAFLD increased 63% for those who gained ≥ 5% of body weight, and decreased 65% for subjects who lost ≥ 5% of body weight [adjusted hazard ration (HR) 1.63, 95% confidence interval (CI), 1.30–2.04 and adjusted HR 0.35, 95% CI, 0.21–0.59, respectively, Table 3]. When the subjects were further divided into 7 groups, the risk of incident NAFLD significantly increased in two groups including weight gain ≥ 10% and 7% ≤ gain < 10% (adjusted HR 2.43, 95% CI, 1.65–3.58 and adjusted HR 1.73, 95% CI, 1.26–2.39, respectively), while the risk of incident NAFLD significantly decreased in the group with -7% < weight loss ≤ -3% (adjusted HR 0.33, 95% CI, 0.22–0.51, Table 3). The HR ratio plot dysplaying the correlation between OBC and risk of incident NAFLD is illustrated in Fig. 1.

**Table 3:** The risk of NAFLD according to overall bodyweight change.

Overall bodyweight change	No. of patients	No. of events	Univariate analysis			Multivariate analysis		
			Hazard ratio (95% CI)	P-value	Overall *P*-value	Hazard ratio (95% CI)	P-value	Overall *P*-value
Per 10% increase	1907	420	1.81 (1.53, 2.14)	< 0.001	< 0.001	2.28 (1.90, 2.74)	< 0.001	< 0.001

Loss ≥ 5%	160	16	0.39 (0.23, 0.64)	< 0.001	< 0.001	0.35 (0.21, 0.59)	< 0.001	< 0.001
Stable weight (within ± 5% change)	1417	288	1 (ref)			1 (ref)		
Gain ≥ 5%	330	116	1.40 (1.13, 1.74)	0.002		1.63 (1.30, 2.04)	< 0.001	

Loss ≥ 10%	24	2	0.28 (0.07, 1.12)	0.07	< 0.001	0.3 (0.07, 1.21)	0.10	< 0.001
10% < loss ≤ 7%	56	7	0.56 (0.26, 1.19)	0.13		0.53 (0.25, 1.13)	0.10	
7% < loss ≤ 3%	254	24	0.31 (0.21, 0.48)	< 0.001		0.33 (0.22, 0.51)	< 0.001	
Stable weight (within ± 3% change)	985	207	1 (ref)			1 (ref)		
3% ≤ gain < 7%	407	98	0.9 (0.71, 1.14)	0.38		1.19 (0.93, 1.52)	0.17	
7% ≤ gain < 10%	112	49	1.69 (1.24, 2.31)	< 0.001		1.73 (1.26, 2.39)	< 0.001	
Gain ≥ 10%	69	33	1.51 (1.04, 2.18)	0.03		2.43 (1.65, 3.58)	< 0.001	

Adjusted for age, sex, hypertension, diabetes, waist circumference, body mass index, triglyceride, high density cholesterol, total cholesterol, alanine transaminase, and number of measurements.

*NAFLD* nonalcoholic fatty liver disease, *CI* confidence interval.

## Discussion

In this study, we demonstrated that overall weight gain of more than 7% significantly increased the risk of incident NAFLD while weight loss of more than 3% reduced the risk of incident NAFLD. Also, overall weight gain was associated with increased risk of incident NAFLD in a dose-dependent manner. However, body weight variability was not associated with the risk of NAFLD. These findings suggest that overall weight change, more than weight variability is associated with the NAFLD development.

Several studies have demonstrated the association between weight loss and the improvement of NAFLD. A 5% or greater weight reduction for one year was associated with ALT improvement and a 3.6-fold increased rate of ALT normalization, suggesting that a 5% reduction in body weight could be recommended as an initial therapeutic target for NAFLD patients^4^. However, one limitation of this study is that an elevated level of transaminase was used as a surrogate marker of NAFLD. A randomized controlled trial showed that a 7% to 10% weight reduction through intensive lifestyle intervention for 48 weeks resulted in improvements in liver chemistry and histology of steatosis, necro-inflammation^19^. The participants in this study were required to have elevated ALT > 41 and BMI ≥ 25 kg/m^2^, and histologically confirmed non-alcoholic steatohepatitis (NASH). Another prospective study reported that greater (≥ 10%) weight loss was associated with the level of improvement in histologic features of NASH including fibrosis, or portal inflammation^4^. A recent study showed that the frequency of NAFLD remission reached a plateau of 43% in subjects with 1–2%/year weight loss^20^. Similar with previous results, in the present study, weight loss between 3 and 7% was associated with a decreased risk of incident NAFLD, suggesting that even a weight loss rate less than 5% may help improve NAFLD. There was no significant association between weight loss more than 7% and the risk of incident NAFLD in this study, which might be due to the small number of patients (24 in the group with loss ≥ 10% and 56 in the group of 10% < loss ≤ 7%, respectively).

Body weight gain is a well-known risk factor of NAFLD. In a prospective study with a 7-year follow-up, weight gain was independently associated with incident NAFLD (odds ratio = 1.14)^21^. Another study found that body weight gain in earlier and later adulthood were all associated with increased risk of NAFLD, with relation to insulin and insulin resistance as key mediators^22^. A large-population study performed among Korean men showed that subjects with weight gain more than 2.3 kg had an approximately 26% higher risk of NAFLD compared to those with stable body weight^23^. However, the quartile was divided based on the absolute value of weight change without taking into account the relative ratio of individual weight change. In this study, overall weight gain was associated with increased risk of incident NAFLD in a dose-dependent manner, as 7% ≤ gain < 10% with aHR 1.73 and gain ≥ 10% with aHR 2.43, respectively.

Although weight loss is commonly recommended as a lifestyle modification in NAFLD patients, weight loss is usually followed by weight gain, leading to weight variability^8^. The effect of body weight variability on clinical prognosis is still controversial^24,25^. Several studies have reported that weight variability was associated with incident diabetes in obese patients^13,26^, and the linking mechanism is suggested as insulin resistance^27^. On the contrary, a community-based prospective cohort study reported that weight variability was a risk factor for abdominal obesity; however, it did not increase the risk of metabolic syndrome^28^. An analysis of Framingham study participants showed that BMI variability was associated with higher risks of getting type 2 diabetes (58%), and getting hypertension (74%) among nonobese participant^29^. However, subjects with high BMI variability were also 163% more likely to get obesity, indicating mixed effects of overall weight gain and weight variability. In addition, a recent study based on the secondary analysis of randomized controlled trial results reported that weight variability did not have significant association with changes in cardio-metabolic risk factors or body composition whereas weight loss improved outcome independent of the degree of variability^30^. Similar with these results, body weight variability per se was not associated with the risk of NAFLD in our study, suggesting that the overall weight change is more important than weight variability in the process of NAFLD development. In this study, the population comprised mostly of lean/non-obese subjects with mean BMI of 22.5 kg/m^2^ at baseline, and laboratory measurements were, on average, within a healthy range. Weight variability is considered as a risk factor for disease specifically in metabolically unhealthy and underweight or overweight/obese populations^31–33^, and therefore effects may be limited in our study.

Recently, a term of “metabolic-associated fatty liver disease (MAFLD)” is proposed in patients with fatty liver^34^. MAFLD include fatty liver patients with other etiologies including alcohol and concomitant liver disease, and exclude NAFLD patients with less than 2 metabolic abnormalities. This term is better to identify population who are at a higher risk of metabolic disease-related outcomes than traditional NAFLD. As we could not evaluate metabolic syndrome components at the follow up time, we could not assess the association of weight variability and incident MAFLD. Further studies are warranted to evaluate the impacts of weight variability on the disease course of MAFLD.

The present study has some limitations. First, we were unable to obtain liver histological samples, the gold standard for the diagnosis of NAFLD. Ultrasonography may produce false-negative results when fatty infiltration of the liver falls below 20%^35^, representing inter- and intra-observer diagnostic variability. In addition, we could not assess the disease severity of NAFLD since ultrasonography cannot differentiate nonalcoholic fatty liver (NAFL) from NASH and noninvasive fibrosis markers or serum ALT at the follow up time were not available in this study. Second, this study’s cohort is a selected population, and may not be representative of the general population. Considering that the study population consisted mainly of relatively healthy subjects, majority of the incident NAFLD cases might present NAFL rather than NASH. Third, although unintentional weight change may be attributed to some underlying diseases, we could not evaluate whether the body weight changes were intentional or unintentional. Lastly, this study lacks information on the dietary habits or physical activity of the participants. Further studies are needed to validate our results.

In conclusion, overall body weight changes rather than bodyweight variability was found to be independently associated with the risk of incident NAFLD. High levels of weight gain more than 7% were significantly associated with incident NAFLD, while 3% to 7% weight loss was associated with decreased risk of incident NAFLD. Such results may have clinical implications for weight loss quantification guidelines for treatment of NAFLD patients.

## Supplementary Information

Supplementary Table S1.
