# Supplementary material for: Body weight gain rather than body weight variability is associated with increased risk of nonalcoholic fatty liver disease
Source: Sci Rep. 2021 Jul 13;11:14428. doi: 10.1038/s41598-021-93883-5 (PMC8277820; doi:10.1038/s41598-021-93883-5)
Supplement: Supplementary file 1 — Supplementary Table S1. [file 41598_2021_93883_MOESM1_ESM.docx]

**Supplementary Table 1. Body weight variability and risk of NAFLD according to overall bodyweight change**

| Overall bodyweight change |  | Univariable analysis | | | Multivariable analysis | | |
| --- | --- | --- | --- | --- | --- | --- | --- |
|  |  | Hazard Ratio (95% CI) | P-value | Overall  P-value | Hazard Ratio (95% CI) | P-value | Overall  P-value |
| Loss ≥ 5% (n=160) | | | | | | | |
| SD | Q4 vs Q1-3 | 2.04 (0.58, 7.2) | 0.27 | 0.27 | 1.17 (0.27, 5.09) | 0.83 | 0.83 |
| CV | Q4 vs Q1-3 | 1.47 (0.47, 4.64) | 0.51 | 0.51 | 0.77 (0.21, 2.86) | 0.70 | 0.70 |
| ARV | Q4 vs Q1-3 | 1.54 (0.55, 4.36) | 0.41 | 0.41 | 1.48 (0.43, 5.04) | 0.53 | 0.53 |
| VIM | Q4 vs Q1-3 | 1.49 (0.47, 4.69) | 0.50 | 0.50 | 0.78 (0.21, 2.9) | 0.71 | 0.72 |
| Stable weight (within ± 5% change) (n=1,417) | | | | | | | |
| SD | Q4 vs Q1-3 | 1.00 (0.72, 1.41) | 0.98 | 0.98 | 0.91 (0.64, 1.29) | 0.58 | 0.58 |
| CV | Q4 vs Q1-3 | 0.63 (0.43, 0.93) | 0.02 | 0.02 | 0.70 (0.47, 1.05) | 0.08 | 0.08 |
| ARV | Q4 vs Q1-3 | 1.14 (0.86, 1.53) | 0.36 | 0.36 | 0.86 (0.63, 1.16) | 0.32 | 0.32 |
| VIM | Q4 vs Q1-3 | 0.72 (0.49, 1.05) | 0.09 | 0.09 | 0.75 (0.51, 1.1) | 0.14 | 0.14 |
| Gain ≥ 5% (n=330) | | | | | | | |
| SD | Q4 vs Q1-3 | 1.68 (1.12, 2.50) | 0.01 | 0.01 | 1.25 (0.81, 1.93) | 0.31 | 0.31 |
| CV | Q4 vs Q1-3 | 1.23 (0.84, 1.81) | 0.28 | 0.28 | 1.21 (0.81, 1.81) | 0.34 | 0.34 |
| ARV | Q4 vs Q1-3 | 1.38 (0.95, 1.99) | 0.09 | 0.09 | 0.88 (0.58, 1.33) | 0.54 | 0.54 |
| VIM | Q4 vs Q1-3 | 1.29 (0.87, 1.90) | 0.20 | 0.20 | 1.09 (0.72, 1.66) | 0.68 | 0.68 |

NAFLD, nonalcoholic fatty liver disease; CI, confidence interval; SD, standard deviation; CV, coefficient of variation, ARV, average real variability; VIM, variability independent of mean; OBC, overall bodyweight change; Q, quartile.

Adjusted for age, sex, hypertension, diabetes, waist circumference, body mass index, triglyceride, high density cholesterol, total cholesterol, alanine transaminase, and number of measurements
